# The Construction of Intelligent Emotional Analysis and Marketing Model of B&B Tourism Consumption Under the Perspective of Behavioral Psychology

**DOI:** 10.3389/fpsyg.2022.904352

**Published:** 2022-05-12

**Authors:** Wenru Guo, Daijian Tang

**Affiliations:** ^1^School of Tourism and Urban-Rural Planning, Zhejiang Gongshang University, Hangzhou, China; ^2^School of Tourism Management, Xinyang Agriculture and Forestry University, Xinyang, China

**Keywords:** behavioral psychology, B&B tourism, sentiment analysis, TPB, marketing model

## Abstract

This manuscript constructs an intelligent sentiment analysis and marketing model for bed and breakfast (B&B) consumption based on a behavioral psychology perspective. Based on the LDA theme model, the theme features and keywords of the reviews covering user feedback are explored from the text data, and the theme framework of user sentiment perception is constructed by combining previous literature on user perception in the B&B market, and the themes of user online reviews are summarized in four dimensions: practical, sensory, cognitive, and emotional components of user experience. In this manuscript, GooSeeker software was selected for data crawling and ROST CM (ROST content mining) developed by Wuhan University was used for text processing. To improve the accuracy of text classification and improve the missing data, the online comment text is divided into sentences by symbols, and the text is divided into words based on sentences, and the spatial vector model and the text feature word weighting method of TF-IDF are used for vector representation, and the polynomial Bayesian classifier is called to identify the topics of sentences. The classical Theory of Planned Behavior (TPB) was used to analyze the influencing factors of the willingness to consume experiential B&B tourism, and countermeasure suggestions for the development of B&B tourism were proposed based on the research findings In the empirical testing stage, a questionnaire on the willingness to consume experiential B&B tourism was designed, and web research was chosen to collect the data. SPSS20.0 was used to conduct reliability analysis, factor analysis, correlation analysis, and regression analysis on the data, and AMOS statistics were used to establish a structural equation model to verify the influence path of willingness to consume experiential B&B tourism. Finally, the moderating path of willingness to consume experiential B&B tourism was verified by using multi-group analysis.

## Introduction

As China’s economy continues to develop and urbanization accelerates, the hustle and bustle of the urban environment have gradually increased the demand for experiential tourism. The increase in tourism demand has driven the expansion of the demand market for experiential tourism and the development of experiential bed and breakfast (B&B) tourism, the main manifestation of which is that B&B tourism as a new form of existence of experiential tourism has been favored by increased consumers (Park et al., 2021). Comprehensive economic data from 2013 to 2019 show that the range of total domestic GDP increase from 2013 to 2019 is 6–8%, and the comprehensive contribution of the tourism industry to GDP also grows from about 7% in 2013 to about 11% in 2019. The tourism accommodation service consumption sector, as one of the comprehensive competitive advantages of tourism destinations, has generally shown characteristics such as stable type and uneven structure (Deng and Lee, 2019). With the gradual increase in the number of experience tourism products, B&B tourism with outstanding experience advantages is also receiving more attention from the government and the community. Along with the development of the domestic B&B industry, national and local governments and industry associations have introduced laws and regulations related to the development of the B&B industry to regulate and encourage the development of B&B (Fusté-Forné and Jamal, 2021).

With the outbreak of the new crown epidemic at the end of 2019, smart tourism has accelerated the reform and innovation of the B&B industry, launching “smart B&B” to realize self-check-in, contactless check-in, and other smart services, thus helping the economic recovery. In addition, the integration of smart home elements into B&Bs has promoted the wisdom of B&B design, network development has promoted the wisdom of B&B marketing, and office automation has promoted the wisdom of B&B management (van Eeden et al., 2020). At present, the development of the B&B industry has a certain scale, and the proportion of the economy in some areas has a certain status, and even become an important initiative to solve the employment problem in some places. Increasingly domestic researchers have started to work on B&B research, and most of the research directions are focused on B&B product development and design, B&B business management, etc.

With the advent of the experience economy, more people love the unique experience. They are tired of the high-rhythm city life of steel and concrete and red lights and wine and are more willing to return to the countryside to experience a different kind of life (Stevens et al., 2019). Look at the stars and explore the wonders of the universe; enjoy the music of the fields under the moon and flowers; feel the magic of the mountains and the water; find harmony in the neighborhood. Living in a B&B, enjoying the beautiful local scenery, tasting the local specialties, feeling the hospitality of the B&B hosts, and participating with the simple laborers is endless fun. With the rise of leisure vacation tourism and self-drive tours, B&Bs have become the first choice for increased city dwellers (Boland et al., 2019). Whether or not they have had the experience of living in the countryside, people always have a beautiful image of the countryside in their hearts. A thousand people have a thousand kinds of countryside imagination. It is these imaginations that attract people to experience and feel the B&B. Compared to standardized star hotels, B&Bs provide more unique and personalized non-standard experiences, such as B&Bs in the design process will be customized according to the local culture or the personal taste of the B&B owner different decoration design style, bringing tourists a unique or different experience, so many B&Bs are favored by tourists (Hartmann et al., 2018).

After nearly 2 years of rapid development, more tourists are becoming increasingly cognizant of B&Bs, and B&Bs are spreading from the very beginning of the Moganshan area to all over the country, and this rapid development has directly pushed B&Bs to the height of competition (Vlaev et al., 2019). However, in some areas, B&Bs are often vacant, and business is not as brisk as expected. B&Bs are ignited by the sentiments of their owners, but they need continuous firewood to stay warm, and this firewood is the source of guests. Therefore, it is extremely important to study the willingness of tourists to stay again, and an in-depth study and interpretation of B&B will better guide the healthy and sustainable development of the B&B industry.

## Related Work

Foreign research on B&B tourism mainly includes research on the B&B tourism industry, B&B tourism enterprise management, and B&B tourism behavior research. Although the concept of “B&B tourism” has not been explicitly proposed in foreign academia, its research objects range from the characteristics of travelers to the operators of B&Bs to the related management system (Kazantzis et al., 2018). In recent years, foreign scholars have focused their research on B&B tourism on the configuration of B&B tourism elements and the marketing of B&B tourism, with Airbnb enabling Internet booking of special B&Bs and being popular among young backpackers for its high-cost performance. The community has 191 countries and offers over 600,000 B&B enthusiasts a choice of different B&B design styles (Kazantzis, 2018). Nielsen K S conducted a comprehensive field study of the British B&B market and summarized the successful experiences of B&B owners. This concept of B&B design has attracted deep recognition from most consumers (Nielsen et al., 2020). Zhang argue that B&B design should be innovative in concept, incorporate new elements of humanity, establish a sense of originality, absorb different types of talents, and pay attention to the quality and service of B&B design to prevent excessive commercialization (Zhang et al., 2019).

In the early 20th century, the American psychologist Hendricker E proposed that behavior is a collection of different bodily responses that an organism uses to adapt to changes in its environment (Hendricker et al., 2018). Mayer D proved through experiments that psychological factors and perception are correlated, and proposed that human psychological factors can influence spatial perception, spatial description, and spatial experience (Mayer and Sulkowski, 2018). Foreign research on behavioral psychology is mainly in spatial design, including plaza design, park design, residential area design, and road design. Yu C C points out that the design of roads, plants, and seating facilities in parks should be based on the psychology of human behavior, as well as the reasonable arrangement of the location of each facility. Another type of research is on the behavioral psychology of the elderly, children, and vulnerable people (Yu et al., 2020). Yang et al. suggest that the design and form of children’s activity places should help children’s growth and psychological development. Research on behavioral psychology of the elderly is mainly explored in the environmental design and age-appropriate design of activity spaces for the elderly (Yang et al., 2018). From the statistical results, the minimum sentiment score is 44.61121 and the maximum is 1641. 655. Aiello analyzed children’s requirements for activity spaces through the theory and methods of child behavioral psychology and applied them to specific practice transformation, summarizing the methods and strategies of children’s activity space design (Aiello et al., 2022). Yang (2019), from the perspective of parent-child interaction, revealed the current situation and problems of parent-child interaction space, combined with the study of behavioral and psychological characteristics of both parties, and increased the interactive design of activity space, aiming to promote parent-child relationship.

While the supply scale of the B&B industry is gradually increasing, tourists are also demanding increasingly personalized and boutique accommodations and the competition in the B&B industry is becoming increasingly intense, and tourists’ willingness to consume B&B tourism has become a key element of competition in the B&B industry. Therefore, this manuscript provides theoretical support for B&B operators in improving their services and enhancing tourists’ consumption intentions by studying the factors and mechanisms influencing tourists’ consumption intentions in experiential B&B tourism.

## Materials and Methods

### Research Design

This manuscript is based on the behavioral psychological perspective to construct an intelligent emotional analysis and marketing model for B&B tourism consumption. Through modeling and analysis of tourists’ behavioral psychology, big data capture tourists’ emotional preferences for B&Bs, to recommend targeted B&Bs that meet their preferences and achieve the purpose of promoting tourists’ tourism consumption and secondary consumption or even word-of-mouth transmission. The influence of the environment on the tourists’ activities in the B&B space is subtle. Through the decoration, color and light source in the space, a return to the original atmosphere can be created, making people feel relaxed and happy.

This manuscript proposes a behavioral psychology B&B tourism consumption intelligent sentiment analysis and marketing model based on the existing literature. By collecting data in the crawl and establishing a structural equation model for empirical analysis, for empirical analysis, SPSS software and AMOS statistical software are used to conduct sample descriptive analysis, reliability analysis, validity analysis, model fit analysis, model correction, and model testing on the data collected from the questionnaire, and the research conclusions are summarized according to the results of the research hypothesis testing.

### Participants

Getting basic information about consumers is the basis of the questionnaire, fully informed of the consumers’ place of origin, gender, age, income status and literacy level, etc. Descriptive statistics were conducted using SPSS 26 software to lay a solid foundation for in-depth investigation and analysis of the consumption situation of B&B. With the development of the online tourism (OTA) business, more tourists are using the online ordering of accommodation products to meet the demand for accommodation in tourist places during their travels (Cormier-MacBurnie et al., 2018). Currently, the OTA platforms with large business volumes in China are mainly Ctrip.com, Meituan, Flying Pig, Yilong, and Where to go. The data used in this study comes from https://i.meituan.com, and the reasons why https://i.meituan.com is chosen as the data source for this study are as follows.

(1) The volume of data about B&B after-sale reviews on https://i.meituan.com is sufficient to meet the demand for a huge volume of data in this study.
(2) Mission. com review data interface is open and friendly, easy to crawl program interface docking and data collection, high collection efficiency, and accurate data.
(3) Compared with Ctrip, Flying Pig, Yilong, and other professional OTA platforms, https://i.meituan.com has a wide range of cross-border. Some tourists only install OTA software before travel and may uninstall the software after travel without reviewing it. However, because Meituan has other businesses that are more closely related to daily life, such as online ordering and group buying, in addition to OTA business, its users are stickier, thus helping to improve the quality of its user reviews. The degree words are descended from the heaviest most degree to the least degree, with a total of 6 levels.
(4) Because of its higher user share and stricter merchant management methods, it is easier to filter the TOP merchants in terms of review volume.

Therefore, the valid reviews on Meituan were selected as the basis of data analysis for the model in this manuscript. The respondents are widely distributed, including tourists from 30 provinces in China, including Jilin, Heilongjiang, Liaoning, Beijing, Tianjin, Shanghai, Inner Mongolia, Shandong, Shanxi, Shaanxi, Yunnan, Sichuan, Qinghai, Guangdong Zhejiang, Hebei, Hunan, Hubei, Jiangsu, Fujian, Gansu, Ningxia, Guangxi, Heilongjiang, Xinjiang, Qinghai, Chongqing, Anhui, Guizhou, and Jiangxi, except Tibet Autonomous Region, and the basic information of tourists shown in Table 1.

**TABLE 1:** Survey of basic information of sampled tourists.

Tourist characteristics	Attributes	Number of samples	Proportion
Sex	Male	299	38.48%
	Female	478	61.52%
Age	A < 18	5	0.64%
	18 ≤ A < 25	80	10.30%
	25 ≤ A < 40	300	38.61%
	40 ≤ A < 60	392	50.45%
	A ≥ 60	0	0.00%
Educational level	Below high school	5	0.64%
	High school	30	3.86%
	College	400	51.48%
	Undergraduate	239	30.76%
	Bachelor degree or above	103	13.26%
Profession	Student	30	3.86%
	Worker	240	30.89%
	Government officer	50	6.44%
	Freelance	150	19.31%
	Individual boss	30	3.86%
	Retirees	203	26.13%
	Others	74	9.52%
Place of residence	First-tier cities	108	13.90%
	Second-tier cities	240	30.89%
	Third- and fourth-tier cities	380	48.91%
	Local	49	6.31%

### Measures

The comments used in this study are mainly labeled comments and text comments, which are collectively referred to as “worded comments” this study. In the design process of the crawler program, in addition to the basic interface docking and B&B ID rotation, the function of automatically excluding the default comments and emoticons is added to the program to ensure the validity of the obtained comments. After the code is run, the corresponding B&B comment data can be crawled. To ensure the readability and organization of the collected reviews, txt files and CSV files are generated and saved, respectively by B&B ID number, and each review is saved with a new line. After eliminating the invalid data such as default positive reviews, the actual sample data of 97,213 valid reviews were obtained, with the shortest reviews being three words, such as “comfortable bed” and “good service”, and the longer reviews being more than 100 words. Further consider the influence of negative words and degree words on the weight, calculate the score of sentiment words in the clause, and then add up the sentiment scores of all the clauses of a comment according to the clause index ID.

Based on the segmentation framework of user comment topics obtained by LDA, the obtained comments were classified into topics. Since the sentences of the obtained online review data are relatively long and usually express perceived value in more than one aspect, the 31130 online reviews are divided into sentences using commas, periods, exclamation marks, and question marks as separators, and multiple separators are split at once by the split function, and the B&B ID is saved as a unique matching primary key for calculating the sentiment of the B&B by topic and by the merchant by topic and by the merchant. After splitting, a total of 165,257 comment clauses are obtained, and the clauses are further cleaned to remove numbers and irrelevant characters.

### Design

Based on the Theory of Rational Behavior (TRA), Ajzen proposed a more refined Theory of Planned Behavior (TPB) in 1985. It was pointed out that behavioral intention is influenced by subjective norms, attitudes, and perceived behavioral control. The theory is widely used in user behavior research, and his main ideas are divided into two parts: first, the size of the probability of consumer behavior is directly determined by purchase intention; second, consumer purchase intention is directly influenced by subjective norms, attitudes and perceived behavioral control, and it is after consumers perceive a product or service that they form attitudes that are emotional and evaluative, and subjective norms are influenced by their preferences (Shalender and Sharma, 2021). In the Internet information era, consumers’ behavioral intentions are also largely influenced by various external online information and ethical and moral values. This manuscript studies consumer behavioral intentions based on online reviews and the theory of planned behavior. The construction of the perceived value evaluation system and the research hypothesis model of B&B is the main content of this chapter. The construction of the perceived value scale is divided into two main stages. Stage 1: Based on the careful reading and analysis of B&B customer reviews, the ROSTCM software was used to conduct word frequency statistics, high-frequency word extraction, and social network analysis of the review content, and the perceived value dimensions were divided in combination with the relevant theoretical bases in the literature to form a preliminary value scale. Phase 2: Based on the results of previous research results and content analysis, questionnaires were designed and distributed, and factor analysis was conducted on the scale to further improve the scale formed in the first phase. Finally, based on the final formed perceived value scale, the research hypothesis on the relationship with behavioral intention is proposed and validated, and management recommendations are made based on the research findings and discussions.

When constructing intelligent sentiment analysis and models for B&B tourism consumption, to ensure that the indicators are scientifically standardized and can truly reflect the requirements that need to be investigated, the indicators need to meet the following principles.

First, the evaluation indicators chosen should be related to the content of the intended B&B tour tourist satisfaction, and have strong representativeness, responding to the main points of tourists’ concerns in the tour as much as possible, and being able to reflect the true relationship objectively and comprehensively between the indicators. Added the function of automatically removing system default comments and emojis in the program. After the code runs, you can crawl the corresponding homestay review data.

Secondly, the whole index system should be set up to include all the factors influencing tourists’ satisfaction and be as comprehensive as possible, and avoid duplication and omission among the factors, errors, and untruths.

Third, design the questionnaire according to the system of indicators established, choosing simple indicators with clear and understandable indicators that are easy to collect.

Fourth, the established index system has a clear hierarchy, i.e., each index has a certain logical relationship with each other, reflecting the characteristics of travel, accommodation, travel, food, tourism, shopping, entertainment, and overall evaluation from different sides, as well as the connection between various aspects. The so-called behavioral psychology is the external action of the ontology after being influenced by the environment (Cheng, 2019). Using the study of the psychology of ontology to achieve the purpose of predicting and inferring people’s behavior, behavior is the direct outward action of human psychological transformation. All the purposeful activities of people are the external manifestation of mental activities become behaviors. Exploring the psychological laws of people in the environmental space, predicting, and guiding the behavioral activities of people in it. In this manuscript, we explore tourists’ consumption intention of B&B tourism from seven aspects: local characteristics, compound environment, local security, transportation, shopkeeper enthusiasm, food and beverage, and consumption level, and the index selection is detailed as shown in Table 2.

**TABLE 2:** Indicators for judging the consumption intention of B&B tourism.

Serial number	Index	Remark
1	Indicators	
2	Local Features	
3	Courtyard environment	
4	Local Security	
5	Transportation	
6	Store owner enthusiasm degree	
7	Dining	
8	Consumption level	

Consumer behavior is a dynamic interaction between consumer perception, emotion, thinking, and behavior. Experience is the behavior of people’s psychological reaction based on external stimuli, usually in the form of changes in emotion as the main expression. Consumer experience is the overall cognition, emotion, and evaluation of the products and services consumed by customers, which is a systematic and comprehensive psychological experience. The split function is used to split multiple separators at one time, and save the homestay ID as the unique matching primary key. The consumption experience runs through the whole consumption process, and its unique economic value is also always reflected in the tourism service (Choe et al., 2021). The experience of B&B tourism consumers can be divided into pre-consumption experience, in-consumption experience, and post-consumption experience by consumption time order. The pre-consumption experience reflects the consumer’s expectations before consumption, which is reflected in the way of travel, schedule, fellow travelers, etc., through a series of searches and inquiries, and the selection of travel by way of strategy. During consumption, experience refers to a series of intuitive evaluations of consumption scene, service, price, etc. Post-consumption experience refers to consumers’ recollection, reminiscence, and evaluation after finishing consumption. The following analysis focuses on the experience evaluation during consumption.

### Analysis

The software used for data crawling in this manuscript is GooSeeker, which is mainly composed of the MS search engine and the DS counting machine. First, in the interface of the MS search engine, the user makes the crawling rules according to his demand for data, including single-page crawling, page-turning crawling, hierarchical crawling, etc. Then, according to the rules written in advance, the software the user can update the data regularly as needed. Finally, the data information is obtained in XML file format and then converted into excel format for later data analysis. The text processing in this manuscript is done with the help of ROST CM (ROST content mining) developed by Wuhan University, which can mine and analyze the text content information, and can-do word separation, high-frequency word extraction, clustering analysis, social network, and semantic network analysis. The software is chosen for this study because of its good support in Chinese and its wide application in academia (Timurkutluk et al., 2021). Since the commenters are very different in terms of region and culture, the comment contents may appear in English, with traditional characters, misspellings, etc. Therefore, before resorting to ROST CM software, the crawled text contents are pre-processed and the text format is converted to TXT. Collect data and establish a structural equation model for empirical analysis. In terms of empirical analysis, SPSS software and AMOS statistical software are used to conduct sample descriptive analysis, reliability analysis, validity analysis, and model fit of the data collected from the questionnaire. Analysis, model revision, model testing, etc., summarize the research conclusions according to the research hypothesis testing results.

The measurement scale consists of three parts: personal statistical characteristics of tourists, a survey scale of consumption intention independent variables, and a survey scale of consumption intention. Personal statistical characteristics include gender, age, personal disposable income, travel experience, family life cycle, etc.; the independent variable survey includes three parts, including consumer product attitudes, consumer social norms, and travel perceptions. Among them, the Consumer product attitudes, consumer social norms, and consumption intention measurement scales draw primarily from the Fishbein and Ajzen proposed the TPB measurement scale.

In this manuscript, Cronbach’s coefficient analysis was used to test the reliability stability of the sample. The scholars have different views on the reliability of the α value, but most of them believe that: α between 0.60 and 0.70 is weak but within the acceptable range; coefficient between 0.70 and 0.80 is quite good; and between 0.80 and 0.90 represents very good reliability. AdaBoost avoids oPF-1717 on the test set. In the test set, there are merchants with low sales of homestay services. RMSE = 19.57, MAE = 17.17 on the training set. On the test set RMSE = 28.21, MAE = 23.57.

The validity test is mainly to verify the reliability of the questionnaire, and the higher the validity of the questionnaire, the higher its reliability. In this study, the validity test will be conducted using the factor analysis method. The initial KMO value and Bartlett’s sphere test are mainly used to determine whether each variable is suitable for factor analysis. When KMO >0.9, it means that it is very suitable for factor analysis, when 0.7 <KMO <0.9, it means that it is suitable for factor analysis; when KMO <0.7, it means that it is not very suitable for factor analysis; when KMO <0.5, it means that it is not suitable for factor analysis. Then the principal component extraction method was used, and the orthogonal rotation method with Kaiser standardization was performed to obtain the rotated component matrix, and the initial eigenvalues >1 were selected as factors.

## Results

### In the Theory of Planned Behavior, Willingness to Consume Refers to the Intensity of an Individual’s Involvement in Particular Consumption Behavior

The main idea of this theory mentions that the individual’s willingness to consume is a direct variable that predicts the individual’s consumption behavior. The actual measurement of the willingness to consume can be measured by three variables: consumer product attitude, consumer social norms, and travel perception, and the three variables have an indirect effect on consumer behavior through the willingness to consume (Nadlifatin et al., 2020). In the field of tourism, consumer willingness to consume is divided into two main dimensions: first, the likelihood of consumers’ first and repeated consumption, and second, consumers’ willingness to publicize after participating in tourism.

### Behavioral Psychological Model Analysis

Since the calculation of the index of spatial vitality of B&B and the index factors of the constituent elements of spatial vitality of B&B are of different magnitudes, the normalized values of such indexes are calculated using the linear function normalization method to better analyze the correlation between the vitality of B&B and the constituent elements of B&B R. The linear function normalization calculation method converts the raw data into a range of values between 0 and 1 (including 0 and 1) by isometric scaling. The equation for the range of values between: Y=no⁢r⁢m(Ymax-Ymin)/(Y+Ymin). Where *Y*_no*rm*_ is the normalization result, Y is the original data set of different scales, Y*_max_* is the maximum value in the original data set, and Y*_min_* is the minimum value in the original data set.

Correlation analysis was conducted separately for the components of spatial vitality of B&B and the vitality index. Correlation analysis refers to the analysis of the closeness of correlation between two different variables and contains the correlation coefficient and the degree of correlation (Aliabadi et al., 2020). Through modeling and analysis of tourists’ behavioral psychology, big data captures tourists’ emotional preferences for homestays, to recommend targeted homestays that meet their preferences, and achieve the purpose of promoting tourists’ travel consumption and secondary consumption, and even word-of-mouth communication. For example, assuming that the vitality index is not correlated with functional density, Pearson’s correlation coefficient (r) tests this hypothesis, and when the hypothesis is correct, if the test produces a result, i.e., a significant p (probability of the result occurring) less than 0.05, then the original hypothesis is not valid, indicating that the vitality index is correlated with functional density is correlated. Conversely, if the result of the test, i.e., the significance p (probability of occurrence of the result), is greater than 0.05, then the original hypothesis is valid and the vitality index is not correlated with functional density.

Since multiple linear regression is a complex analysis method, high correlation among predictor variables may create the problem of multicollinearity, which results in instability in the estimation of predictor variables in the regression equation. The larger the number of variables, the more complex the analysis will be, and the smaller the number, the more information will be obtained. Therefore, the variables are first subjected to principal component analysis to reassign and combine them into a new and mutually unrelated set of composite variables. Principal component analysis converts a set of potentially correlated variables into a set of intrinsically uncorrelated variables using orthogonal transformation, and the transformed variables are called principal components. First, factor analysis is performed on nine elements related to vibrancy: billboard density, seating density, shelter density, lighting facility density, the width of the street, interface transparency, storefront density, business mix, and social place density. Understand the correlation between each element. As one of the comprehensive competitive advantages of tourism destinations, the field of tourism accommodation service consumption generally shows the characteristics of stable type and uneven structure.

### Intelligent Sentiment Analysis and Model Construction for Bed and Breakfast (B&B) Tourism Consumption

**FIGURE 1:**
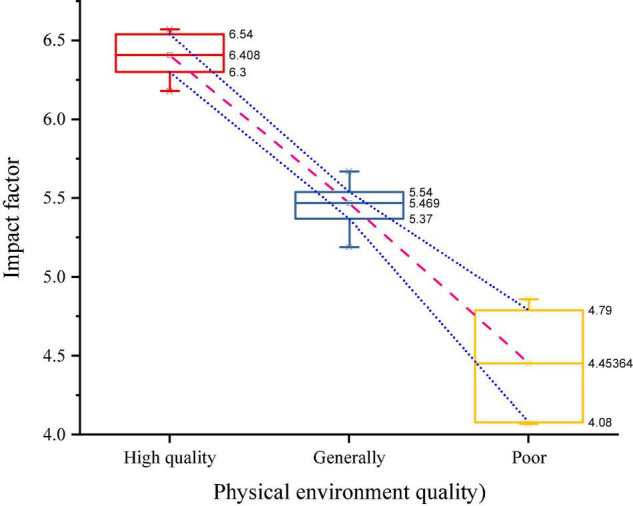
Relationship between environmental quality and different activities.

The better the quality of the environment will directly affect human interaction activities, the better the quality of the environment, the higher the frequency of spontaneous and social activities will occur, especially the frequency of spontaneous activities is completely influenced by the surrounding environment in which they are located. The environment influences human behavior, and communication increases the longer people stay in open public spaces. The influence of the environment on the travelers’ activities in the B&B space is subtle, through the decoration, color, and light source in the space can create a back-to-basics mood, making people happy and relaxed, but also the use of space division to influence the behavior of travelers’ activities, increasing the length of people’s activities in the B&B, making the space more habitable and fuller of life, as shown in Figure 1.

The dictionaries selected in this manuscript include the sentiment lexicon of Zhiwang and the Bosen sentiment dictionary, which can be divided into three major categories according to their functional types, namely sentiment words, degree words, and negation words. Since the sentiment lexicon on the Internet does not assign weights to sentiment words, this manuscript assigns a weight of 1 to each positive sentiment word and a weight of −1 to each negative sentiment word and assumes that the sentiment values satisfy the linear superposition principle. The sentiment words include positive evaluation words, positive sentiment words, negative evaluation words, and negative sentiment words. The degree words are lowered from the heaviest degree of most to least degree in order, with 6 levels. Negative words include words such as no, no, and do not. Negative words are used multiple times in sentiment analysis as a negative expression of affirmation. The launch of “smart homestays” to realize self-service check-in, the arrival of the era of smart homestays, and the realization of smart services such as contactless check-in to help economic recovery. The sentiment words from the Zhiwang dictionary and the Bosen dictionary were combined, and sentiment words with opposite and repeated sentiment polarity were eliminated.

The sentiment score is calculated by first splitting the text into clauses, taking the clause as the smallest analysis unit, then splitting and de-stopping the clauses, and then matching them according to the sentiment dictionary to get the sentiment weight of the clauses. The sentiment scores of the sentiment words in the sentences were calculated by considering the influence of negation and degree words on the weights, and then the sentiment scores of all sentences of a review were summed up according to the sentence index IDs.

Output sentence sentiment tendency based on sentiment score. For a clause comment of a B&B user S_1,_S_2,_…,S*_m_*. a single sentence, suppose *w*_*j*_(*j* = 1,2…*n*) is one of the sentiment words of a sentence *S*_*i*,_ and *S_wj_* is the weight of this sentiment word. When out now the degree adverb *w*_*a*_ modifies the emotion *w*_*j*_, the weight of the emotion *w*_*j*_ is adjusted to:


(1) $$O_{w_{j}}=M_{w_{a}}\times S_{w_{j}}$$

**FIGURE 2:**
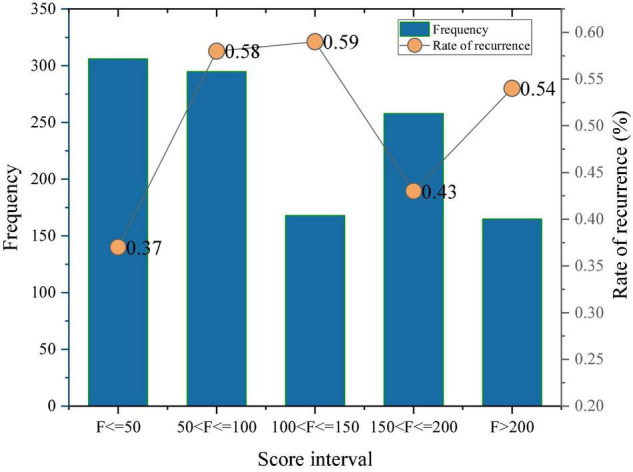
Statistical results of the sentiment score of B&B.

Look at the starry sky and explore the mysteries of the universe; enjoy the music in the fields quietly under the moonlight before the flowers; feel the magic of the mountains and rivers; Living in a homestay, enjoying the beautiful local scenery, tasting local specialties, feeling the hospitality of the homestay owner, and participating in it with the simple laborers, it is a lot of fun. Employing sentiment dictionary matching, the sentiment tendency values of all clauses can be obtained. Since sentiment sentences are generally short, there are cases where the sentiment score of a sentence is not matched to a sentiment word, which affects the calculation of the sentiment score of the total review, so these sentiment sentences need to be removed. The sentiment score of B&B merchants was calculated by simply grouping and summing the sentiment scores of B&B merchants according to their merchant IDs. The difference in sentiment scores is not only due to the sentiment tendency expressed by the reviews on the platform, but also the number of B&B reviews is an important factor in the sentiment scores (Figure 2).

### Intelligent Emotional Analysis and Marketing Model Implementation of Bed and Breakfast (B&B) Consumption Based on Behavioral Psychology

**FIGURE 3:**
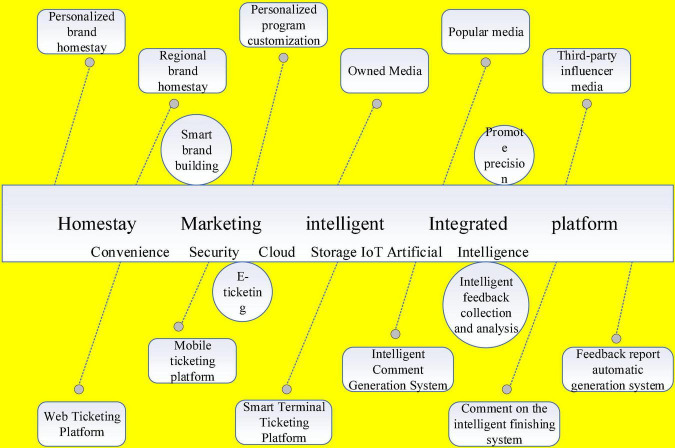
B&B marketing index system.

In addition to solving the problem of ordering B&B products, e-ticketing also enables room price positioning. The strategy of B&B room rates follows a variety of factors such as low and high seasons and casual guests for floating pricing, and off-season or certain special times, price reduction promotions can be used to attract guests to achieve occupancy rates. The current e-ticketing service is different from the traditional ticketing method, it realizes paperless, electronic, and systematic, and travelers can make online reservations to buy B&B products through WeChat, support treasure, Taobao, and Ctrip website; and the price positioning refers to the possibility of adjusting B&B room prices according to holidays, analyzing the price changes of neighboring B&Bs in real-time, and taking competitive strategies. The implementation of e-ticketing not only changes the ordering method of B&Bs and increases sales, but also improves the efficiency of managers, provides consumers with a more convenient and fast way to consume, and shows the “wisdom” of B&B marketing, and the B&B marketing index system is shown in Figure 3.

**FIGURE 4:**
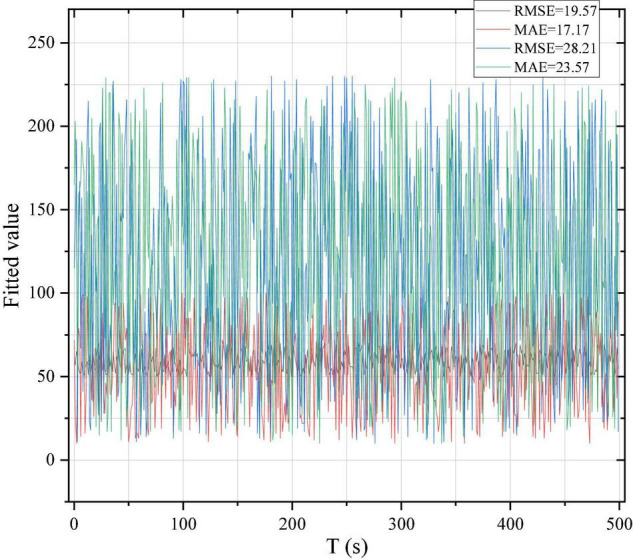
Model fitting effect.

AdaBoost is an integrated algorithm based on boosting, which is based on the idea of building different classifiers for the same training set, with four common parameters including the type of base learner’s base_estimators, error type loss weak learners’ iterations n_estimators, and learning_rate. According to the results of the tuning parameters, the settings are based on the regression. The tree is used as the base model, the maximum depth of the tree max_depth = 15, the number of iterations of the weak classifier n_estimators = 150. r3 = 0.54 for AdaBoost on the training set and r3 = 0.36 on the test set. from plotting the 801 B&Bs in order, AdaBoost on the test set is a good choice for using the corpse purchase taboo of- 1717. 1717. in the test, the set is the merchant with low sales of B&B services. On the training set RMSE = 19.57 and MAE = 17.17. On the test set RMSE = 28.21 and MAE = 23.57, as shown in Figure 4.

## Discussion

At the same time, the initially perceived value dimensions and measurement scales for the special scenario of B&B were formed by drawing on the summaries of the existing related research results, and then a validation factor analysis was conducted on the survey sample data, finally concluding that the five dimensions constitute the customer perceived value of B&B, with a cumulative explanation of 73.499 variance values, which can better reflect the content of the customer perceived value of B&B. The conclusion is that five dimensions constitute the customer perceived value of B&Bs, explaining 73.499 variables, which can better reflect the content of customer perceived value of B&Bs. In terms of customer segmentation, there were no significant differences in the perceptions of the five variables of perceived value among customers of different genders, ages, and education levels. The analysis of the data in this manuscript shows that the target market of B&Bs is mostly highly educated and young, which is consistent with the study of Chen L. et al. In the new consumer era, B&Bs have long been more than simple accommodation and reception facilities; they have become a place for people’s emotional support and the pursuit of inner peace, which has its unique flavor. Consumer demand is more diversified, more focused on spiritual pleasure and personality realization.

## Conclusion

This manuscript integrates the disciplines of management and computer science, uses data analysis techniques and content analysis methods, sentiment analysis methods, and other research methods, takes the comments on the Meituan platform as the object of analysis, carries out word separation and word frequency statistics on the comment text with the help of ROST CM, uses the space vector model and TF-IDF text feature word weighting method to carry out vector representation, uses sentiment analysis techniques to The sentiment analysis technique was used to study the emotional image of the experiential elements in the review text. SPSS20.0 was used to conduct reliability analysis, factor analysis, correlation analysis, and regression analysis on the data, and AMOS statistics were used to establish a structural equation model to validate the influence path of the willingness to consume experiential B&B tourism. The competition in the homestay industry is becoming increasingly intense, and tourists’ willingness to spend on homestay tourism has become a key factor in the competition in the homestay industry. The results show that this model can obtain the key indicators of tourists’ B&B consumption intention through big data analysis. It has the significance to promote the improvement of B&B tourism consumption and marketing. The study shows that the most important influencing factor that drives B&B customer satisfaction is the emotional value in perceived value, and the degree of influence is higher than other value dimensions, and B&B operators should tie in this breakthrough to enhance customer perception of emotional value, thus improving customer satisfaction.

## Data Availability Statement

The raw data supporting the conclusions of this article will be made available by the authors, without undue reservation.

## Author Contributions

WG and DT collaborated on the manuscript. Both authors contributed to the article and approved the submitted version.

## Conflict of Interest

The authors declare that the research was conducted in the absence of any commercial or financial relationships that could be construed as a potential conflict of interest.

## Publisher’s Note

All claims expressed in this article are solely those of the authors and do not necessarily represent those of their affiliated organizations, or those of the publisher, the editors and the reviewers. Any product that may be evaluated in this article, or claim that may be made by its manufacturer, is not guaranteed or endorsed by the publisher.

## References

[B1] Aiello F. Bonanno G. Foglia F. (2022). On the choice of accommodation type at the time of Covid-19. Some evidence from the Italian tourism sector. *Curr. Issues Tour.* 25 41–45. 10.1080/13683500.2020.1846504

[B2] Aliabadi V. Gholamrezai S. Ataei P. (2020). Rural people’s intention to adopt sustainable water management by rainwater harvesting practices: application of TPB and HBM models. *Water Supply* 20 1847–1861. 10.2166/ws.2020.094

[B3] Boland D. H. Juntunen C. L. Kim H. Y. Adams E. M. Navarro R. L. (2019). Integrated behavioral health curriculum in counseling psychology training programs. *Couns. Psychol.* 47 1012–1036. 10.1177/0011000019895293

[B4] Cheng E. W. L. (2019). Choosing between the theory of planned behavior (TPB) and the technology acceptance model (TAM). *Educ. Technol. Res. Dev.* 67 21–37. 10.1007/s11423-018-9598-6

[B5] Choe J. Y. Kim J. J. Hwang J. (2021). Innovative marketing strategies for the successful construction of drone food delivery services: merging TAM with TPB. *J.Travel Tour. Mark.* 38 16–30. 10.1080/10548408.2020.1862023

[B6] Cormier-MacBurnie P. Mombourquette P. Sneddon G. Young G. (2018). The B&B sector in nova scotia: some preliminary evidence from TripAdvisor reviews. *Small Bus. Inst. J.* 14 41–60.

[B7] Deng Y. T. Lee H. (2019). Exploring the dimensions of bed and breakfast (B&B) visitors’ experiences. *Int. J. Tour. Sci.* 19 166–180. 10.1080/15980634.2019.1663989

[B8] Fusté-Forné F. Jamal T. (2021). Co-creating new directions for service robots in hospitality and tourism. *Tour. Hosp.* 2 43–61. 10.3390/tourhosp2010003

[B9] Hartmann W. E. St Arnault D. M. Gone J. P. (2018). A return to “the clinic” for community psychology: lessons from a clinical ethnography in urban american indian behavioral health. *Am. J. Commun. Psychol.* 61 62–75. 10.1002/ajcp.12212 29266300

[B10] Hendricker E. Bender S. L. Ouye J. (2018). Family involvement in school-based behavioral screening: a review of six school psychology journals from 2004 to 2014. *Contemp. Sch. Psychol.* 22 344–354. 10.1007/s40688-017-0163-9

[B11] Kazantzis N. (2018). Introduction to the special issue on processes of cognitive behavioral therapy: does “necessary, but not sufficient” still capture it?. *Cogn. Ther. Res.* 42 115–120. 10.1007/s10608-018-9891-z

[B12] Kazantzis N. Luong H. K. Usatoff A. S. Impala T. Yew R. Y. Hofmann S. G. (2018). The processes of cognitive behavioral therapy: a review of meta-analyses. *Cogn. Ther. Res.* 42 349–357. 10.1007/s10608-018-9920-y

[B13] Mayer D. Sulkowski A. (2018). The US constitution’s emoluments clauses: how history, behavioral psychology, and the framers’ understanding of corruption all require an end to president trump’s conflicts of interest. *Br. J. Am. Leg. Stud.* 7 257–289. 10.2478/bjals-2018-0010

[B14] Nadlifatin R. Miraja B. Persada S. Belgiawan P. F. Redi A. P. Lin S.-C. (2020). The measurement of University students’ intention to use blended learning system through technology acceptance model (TAM) and theory of planned behavior (TPB) at developed and developing regions: lessons learned from Taiwan and Indonesia. *Int. J. Emerg. Technol. Learn.* 15 219–230. 10.3991/ijet.v15i09.11517

[B15] Nielsen K. S. van der Linden S. Stern P. C. (2020). How behavioral interventions can reduce the climate impact of energy use. *Joule* 4 1613–1616. 10.3390/ijerph19020853 35055675 PMC8775624

[B16] Park I. J. Yun D. Kim P. B. Hai S. (2021). How to fuel hotel employees’ daily innovative work? The interplay of daily affect and career future time perspective. *J. Hosp. Mark. Manag.* 30 759–783.

[B17] Shalender K. Sharma N. (2021). Using extended theory of planned behaviour (TPB) to predict adoption intention of electric vehicles in India. *Environ. Dev. Sustain.* 23 665–681. 10.1007/s10668-020-00602-7

[B18] Stevens M. W. R. King D. L. Dorstyn D. Delfabbro P. H. (2019). Cognitive–behavioral therapy for Internet gaming disorder: a systematic review and meta-analysis. *Clin. Psychol. Psychother.* 26 191–203. 10.1002/cpp.2341 30341981

[B19] Timurkutluk B. Altan T. Toros S. Genc O. Celik S. Korkmaz H. G. (2021). Engineering solid oxide fuel cell electrode microstructure by a micro-modeling tool based on estimation of TPB length. *Int. J. Hydrog. Energy* 46 13298–13317. 10.1016/j.ijhydene.2021.01.165

[B20] van Eeden L. M. Slagle K. Crowther M. S. Dickman C. R. Newsome T. M. (2020). Linking social identity, risk perception, and behavioral psychology to understand predator management by livestock producers. *Restor. Ecol.* 28 902–910. 10.1111/rec.13154

[B21] Vlaev I. King D. Darzi A. Dolan P. (2019). Changing health behaviors using financial incentives: a review from behavioral economics. *BMC Public Health* 19:1059. 10.1186/s12889-019-7407-8 31391010 PMC6686221

[B22] Yang C. H. Chou Y. C. Hou J. S. Thac D. V. Huang C.-C. Lu C. H. (2018). Analyzing the optimal level of biotope quality and cost planning for sustainable development in regional tourism: study of B&B houses in Taiwan. *Int. J. Bus. Econ.* 17 73–94.

[B23] Yang R. (2019). “Research on the B&B (Bed and Breakfast) Market Network Attention in China Based on Baidu Index,” in *Proceedings of the 2019 2nd International Workshop on Advances in Social Sciences (IWASS 2019)* (London: Francis Academic Press), 37–44.

[B24] Yu C. C. Liu C. C. Chang M. Y. (2020). Research on the key factors of the success of the theme B&B business strategy. *Adv. Manag. Appl. Econ.* 10 1–9. 10.5171/2016.236549

[B25] Zhang H. Z. Yu H. Xu Y. C. Zheng J. X. Lu L. (2019). Analysis on the characteristics of Minsu (B&B) research in Taiwan, China and the theoretical framework of Minsu (Homestay Inn) research in Mainland China. *Tour. Tribune* 34 95–111. 10.1186/s12868-016-0283-6 27534393 PMC5001212

